# Selective venous sampling in primary hyperparathyroidism caused by ectopic parathyroid gland: a case report and literature review

**DOI:** 10.1186/s12902-023-01376-5

**Published:** 2023-07-06

**Authors:** Xubin Yang, Xueyan Chen, Huan Xu, Junwei Chen, Bin Yao, Qiongyan Lin, Hongrong Deng, Wen Xu

**Affiliations:** 1 Department of Endocrinology and Metabolic Diseases, Guangdong Provincial Key Laboratory of Diabetology, the Third Affiliated Hospital of Sun Yat-Sen University, No. 600 Tianhe Road, Guangzhou, 510630 Guangdong Province China; 2 Department of Interventional Radiology, the Third Affiliated Hospital of Sun Yat-Sen University, Guangzhou, Guangdong Province China; 3 Department of Endocrinology, Jieyang People’s Hospital (Jieyang Affiliated Hospital, Sun Yat-Sen University), Jieyang, Guangdong China

**Keywords:** pHPT, PTH, Ectopic parathyroid gland, SVS, Case report

## Abstract

**Background:**

As an invasive technique, selective venous sampling (SVS) is considered a useful method to identify a lesion’s location to increase the success rate of secondary surgery in patients with primary hyperparathyroidism (pHPT) caused by ectopic parathyroid adenomas.

**Case presentation:**

We present a case of post-surgical persistent hypercalcemia and elevated parathyroid hormone (PTH) levels in a 44-year-old woman with previously undetected parathyroid adenoma. An SVS was then performed for further localization of the adenoma, as other non-invasive methods showed negative results. After SVS, an ectopic adenoma was suspected in the sheath of the left carotid artery, previously considered as a schwannoma, and was pathologically confirmed after the second operation. Postoperatively, the patient’s symptoms disappeared and serum levels of PTH and calcium normalized.

**Conclusions:**

SVS can provide precise diagnosis and accurate positioning before re-operation in patients with pHPT.

## Background

Primary hyperparathyroidism (pHPT) is a common endocrine disease caused by improper secretion of parathyroid hormone (PTH), leading to increased serum calcium levels [1]. The causes of pHPT include a single adenoma, multiple adenomas, diffuse hyperplasia, and carcinoma [1]. The main treatment for pHPT is surgery. However, after surgery, some patients may still have the persistently high calcium and require a second operation, wherein the difficulty of the re-operation significantly increases [2]. There are also several postoperative patients who are eventually diagnosed with ectopic parathyroid adenomas that were previously diagnosed as parathyroid adenomas and operated upon [3–6]. Combined with the influence of the first operation, these all make the second operation more challenging. Therefore, more accurate diagnosis and precise localization of the lesion are paramount to increase the success rate of the second surgery.

Herein, we report a case of persistent hyperparathyroidism in a patient who continued to experience hypercalcemia after parathyroidectomy. After selective venous sampling (SVS), an ectopic parathyroid gland was finally identified. Subsequently, a second operation was performed and the mass was pathologically confirmed as an ectopic parathyroid adenoma.

## Case presentation

**Fig. 1:**
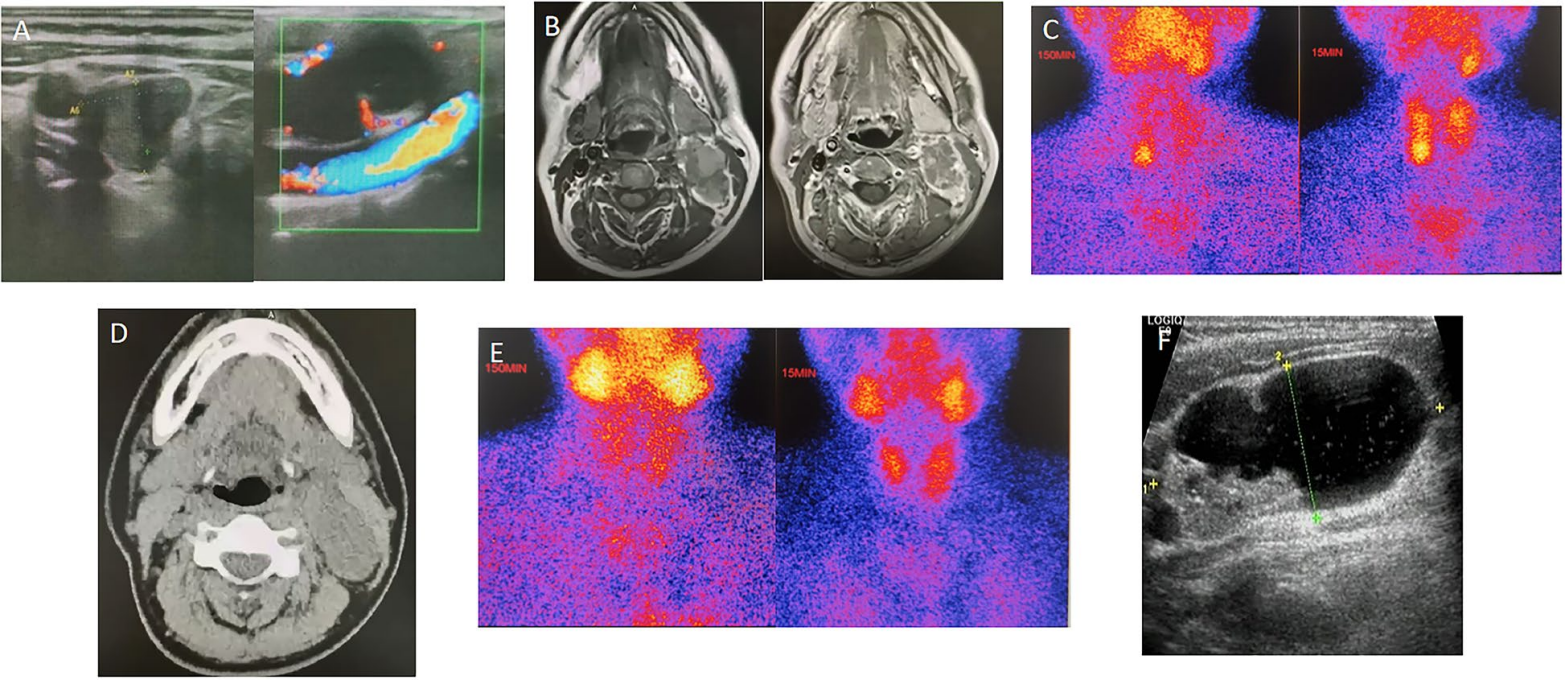
US showed a mass close to the lower side of the right thyroid lobe and a cystic solid mass in the sheath of the left carotid artery (**A**); MRI showed a cystic solid mass in the sheath of the left carotid artery, about 48 mmX25 mmX39 mm in size, with clear boundary. The solid part showed slightly low signal intensity on T1WI and slightly high signal intensity on T2WI, and the cystic part showed uniform high signal intensity on T1WI and T2WI (**B**); MIBI showed an uptake in the right inferior parathyroid mass (**C**); CT revealed a 46 mmx23 mm mass in the sheath of the left carotid artery (**D**); The postoperative MIBI examination was negative (**E**); The neck US image of the left neck mass (**F**)

A 44-year-old woman with symptoms of nausea, vomiting, and weakness of the limbs for more than 10 months was admitted to a local hospital. Initial examination revealed high blood pressure; the systolic blood pressure exceeded 200 mmHg. Laboratory investigations showed that the patient had a total serum calcium of 4.84 mmol/L (reference range: 2.1-2.6 mmol/L) and a PTH level of 1396.5 pg/mL (reference range: 12.00–88.00 pg/mL). She was diagnosed with pHPT according to the guidelines for diagnosis and treatment by Marcella D Walker and Shonni J Silverberg [1]. Preoperative neck ultrasonography (US) revealed a mass close to the lower side of the right thyroid lobe that was considering as right parathyroid adenoma and a cystic solid mass in the sheath of the left carotid artery that was considered as schwannoma by the radiologist, which was consistent with magnetic resonance imaging (MRI) (Fig. 1A, B). ^99m^Tc-Sestamibi scintigraphy (MIBI) (Fig. 1C) showed an uptake in the right inferior parathyroid mass; therefore, the patient was suspected of having right lower parathyroid adenoma. However, MIBI was negative in cystic solid mass in the sheath of the left carotid artery.

**Fig. 2:**
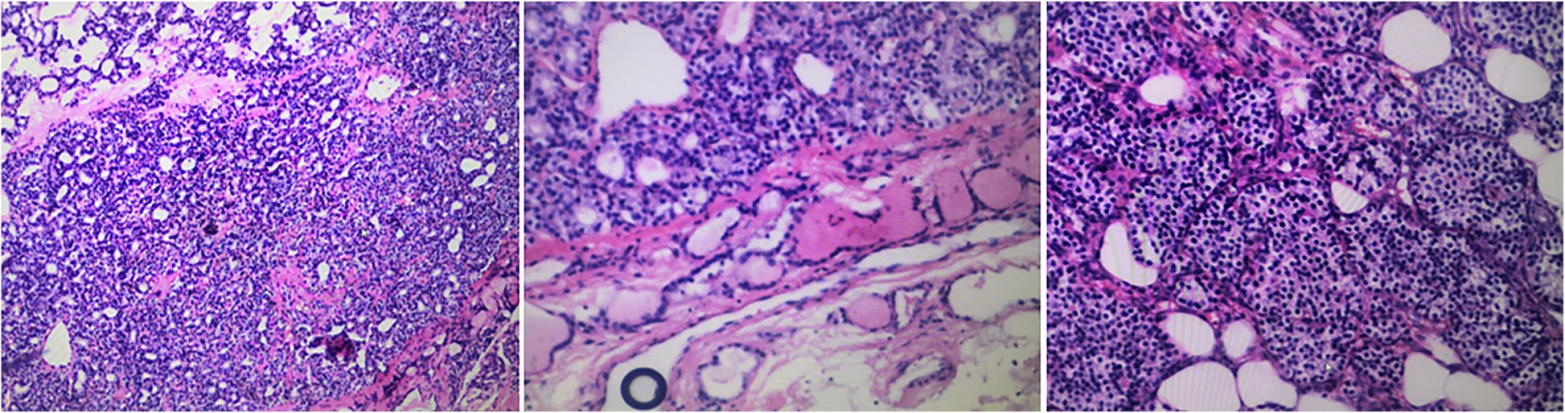
The pathology after the first operation

**Fig. 3:**
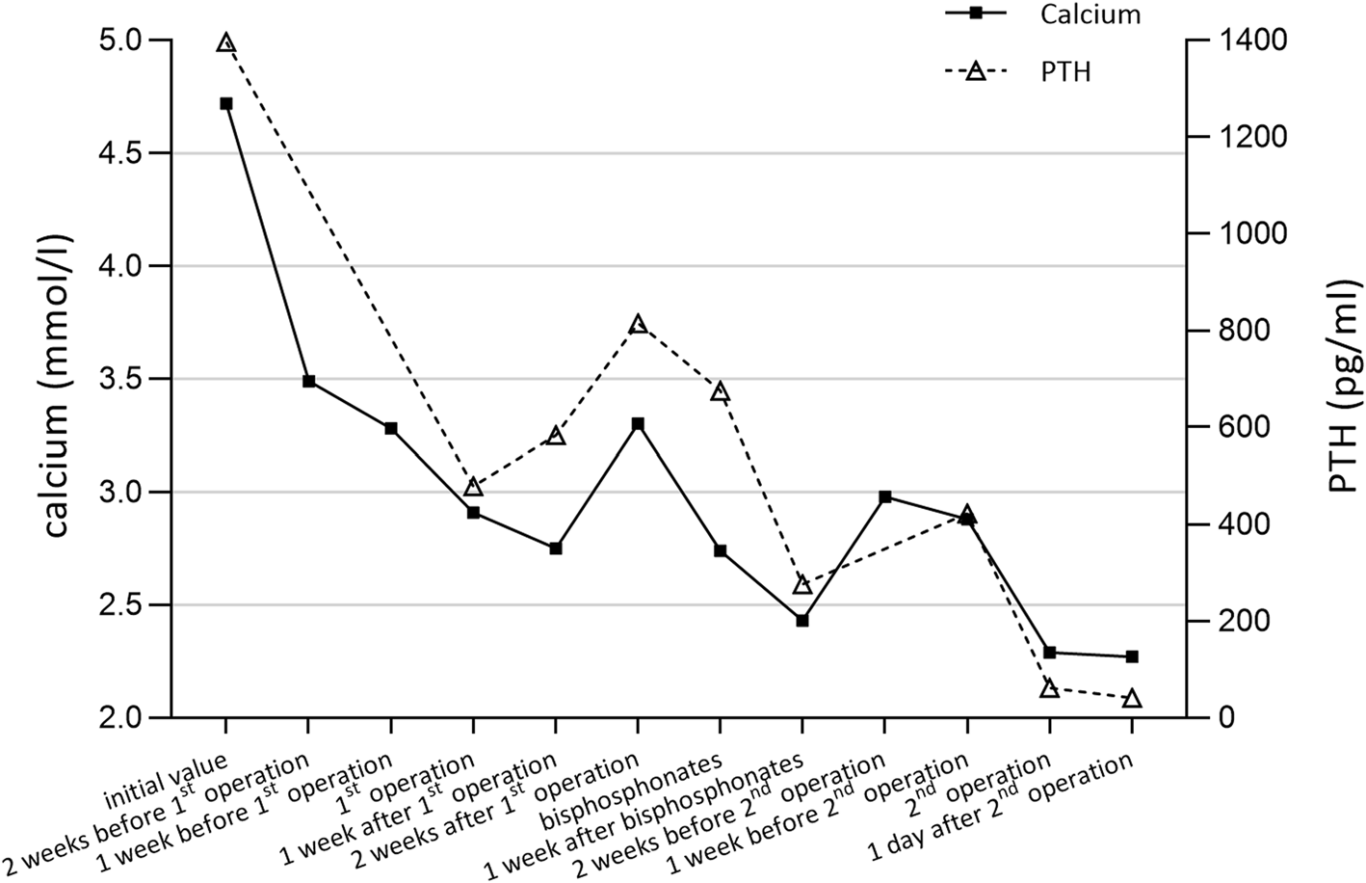
Changes of PTH and serum calcium levels in this patient

The patient was diagnosed with pHPT and was treated with intravenous hydration with 0.9% saline daily and diuretics. The mass and the tissue of the right lower pole of the thyroid gland were removed after operation. Intraoperative quick-frozen section showed the possibility of thyroid adenoma nodule. Meanwhile, intra-operative PTH level did not show significant decrease. Considering that frozen section analysis and intraoperative PTH measurement could not always provide the final definitive diagnosis and further neck exploration would cause unnecessarily extra damage and prolong the operation time, the operation was terminated after communicating with the family members by the surgeons at the local hospital. Finally, the paraffin-embedded tissue was showed to be a thyroid follicular adenoma with a small amount of parathyroid tissue on postoperative pathologic examination (Fig. 2). After surgery, the patient’s symptoms persisted without a significant decrease in PTH level (range: 276.30–615.2 pg/mL) and serum calcium (range: 2.43–3.24 mmol/L) (Fig. 3). Subsequently, computed tomography (CT) reported a mass in the sheath of the left carotid artery, while the MIBI findings were still negative (Fig. 1D, E). Even positron emission tomography (PET) did not show increased metabolism of the left neck mass which was considered a neurogenic tumor, highly suggestive of the schwannoma suggested by the radiologist. During that time, the patient was treated with bisphosphonates – zoledronic acid. However, serum calcium was still higher than the normal range during treatment.

**Fig. 4:**
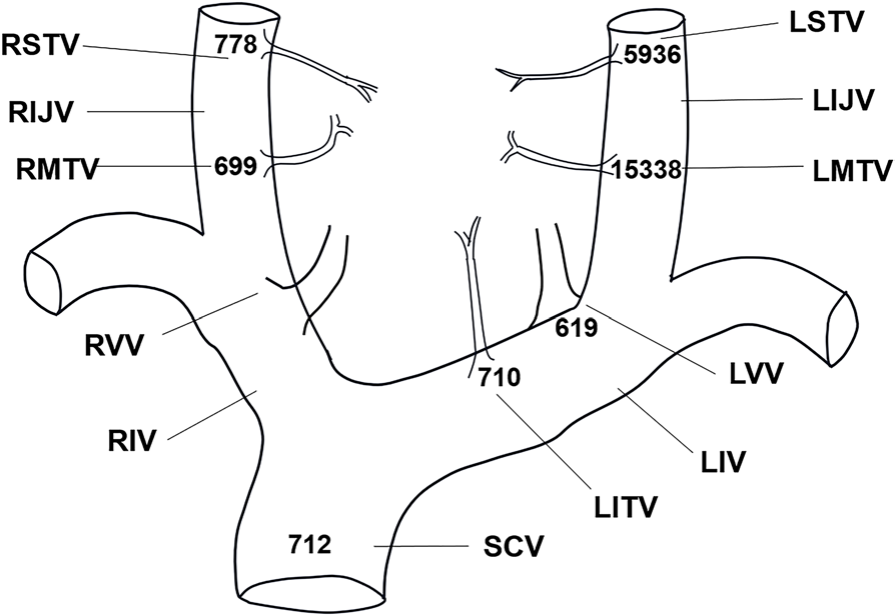
Results of PTH levels (pg/ml) during SVS. SCV: superior caval vein; RIJV: right internal jugular vein; LIJV: left internal jugular vein; RVV: right vertebral vein; LVV: left vertebral vein; RIV: right innominate vein; LIV: left innominate vein; RSTV: right superior thyroid vein; LSTV: left superior thyroid vein; RMTV: right middle thyroid vein; LMTV: left middle thyroid vein; LITV: left inferior thyroid vein

**Fig. 5:**
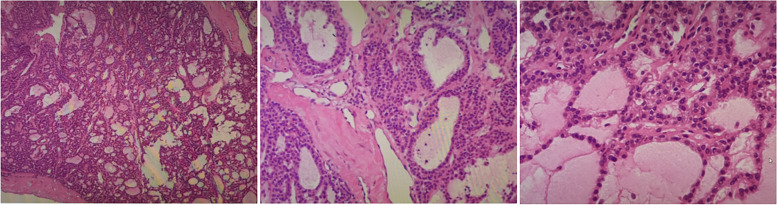
The pathology after the second operation

Later, the patient was admitted to our hospital for further diagnosis and treatment. Upon admission to our hospital, a more detailed history was obtained. Her family and other medical histories were unremarkable. On physical examination, her blood pressure was slightly high (BP: 151/101 mmHg); a 4-cm–long, old, horizontal surgical scar could be seen on the neck, which was well healed. A mass was palpable behind the sternocleidomastoid muscle of the left neck, with smooth surface, clear borders, normal mobility and no tenderness. Laboratory investigations in our hospital showed hypercalcemia and elevated PTH level. In addition, the patient also had hypokalemia (2.8 mmol/L, reference range: 3.5–5.3 mmol/L), increased urinary calcium excretion (7.9 mmol/24 h, reference range: 2.5–7.5 mmol/24 h), and decreased urinary phosphorus excretion (13.5 mmol/24 h, reference range: 16.1–42 mmol/24 h). Osteoporosis was defined by dual X-ray absorptiometry (DXA) (T score: -2.5 or below), and the bone mineral density (BMD) values (in g/cm^2^) of the lumbar spine (L1–L4), femoral neck, and hip were 0.731, 0.559, and 0.694, respectively. T-scores were -2.9, -2.6, and -2.0, respectively. US of the neck also suggested that the mass in the sheath of the left carotid artery might be a schwannoma (Fig. 1F). To identify the exact lesion location, an SVS was performed. Following SVS, the highest quick PTH peaks in the left middle and superior thyroid vein were identified (Fig. 4). This result highly suggested that a PTH-producing tissue was present in the cystic solid mass in the sheath of the left carotid artery, which was suggestive of an ectopic parathyroid gland. The patient then underwent a second operation to remove the mass in the sheath of the left carotid artery; the excised mass measured 5.5 cm × 2.5 cm × 2 cm in size. The final pathological diagnosis of an ectopic parathyroid adenoma was confirmed (Fig. 5). On the first day after the second operation, the patient’s serum calcium and PTH levels had normalized. Her symptoms of nausea, vomiting, and weakness also disappeared postoperatively. She was pleased with the treatment.

## Discussion

PHPT is a common endocrine disease characterized by elevated or inappropriate secretion of serum PTH level and increased serum calcium [1, 7, 8]. The most common cause of pHPT is single parathyroid adenoma, which accounts for 80% of all cases [9]. Rarely, it can be caused by parathyroid carcinoma or parathyroid cyst [10]. The first line treatment for pHPT is resection of the lesion. However, precise identification of the lesion location, which can contribute to the increased rate of successful surgery, is still a clinical challenge. In clinical practice, there are many methods to detect and locate hyperactive parathyroid tissue, such as neck US, MIBI, CT, MRI, and SVS [11]. In our patient, the cause of most symptoms and laboratory results were preliminarily attributed to the excessive secretion of PTH in the right lower parathyroid adenoma with corresponding results of neck US, MRI, and MIBI in the local hospital. Generally, the only definitive treatment for pHPT is surgical removal of the lesion, although some drugs such as bisphosphonates have shown some effects [12]. Therefore, this patient subsequently underwent surgery in the local hospital. During the operation, the frozen section analysis indicated thyroid tissue rather than parathyroid tissue and the intraoperative PTH measurement showed no significant decrease. Considering frozen section analysis and intraoperative PTH measurement could not always provide the final definitive diagnosis [13, 14], the surgeons at the local hospital decided not to perform further neck exploration and terminate the operation to avoid unnecessary damage and prolonging the operation time after communicating with the patient's family members. However, her serum calcium and PTH levels were still abnormal within 6 months after the surgery, which is when persistent pHPT (P-pHPT) was confirmed [15]. This process highlights the need for more precise preoperative localization and cautious surgery, especially when there are other masses present. In such cases, the involvement of internal medicine physicians in the Multi-Disciplinary Treatment (MDT) is also needed. Among patients with pHPT, previous studies showed that the persistence or recurrence of the condition occured in 2.5–5% of cases [16, 17]. The reasons for P-pHPT may include inexperienced surgeons, inaccurate imaging, and ectopic parathyroids [15, 18]. In this case, a second operation was needed that would inevitably experience lower success rate than the primary operation [19]; additionally, the second operation is typically more difficult and challenging, given the long operation time and high risk of complications [2]. Precise preoperative lesion identification examinations are required and are paramount to improve the success rate of reoperation.

### Non-invasive methods for parathyroid lesion localization

In approximately 90% patients with pHPT who have not undergone surgery, the lesion location can be accurately identified through non-invasive examinations. Since the methods before surgery are important for positioning, we compared various methods by reviewing literature. Being a simple and inexpensive method, US is often used as a first-choice imaging examination, with a sensitivity of 89% [20]. However, it is also limited by gland size and the sonographer’s experience. Some studies have shown that US is often not suitable for patients with ectopic parathyroid glands and those due for a second operation [21]. MIBI, which has a sensitivity of 68–86% and a specificity of 90% [22, 23] before initial surgery, has also been shown to have many limitations [24–27]. MIBI could not detect small lesions, hyperplasia, and minor changes in multiple parathyroid glands. The low sensitivity [28] of MIBI prior to re-surgery for P-pHPT was observed in patients with small size of the remaining glands, hyperplasia, and anatomical changes resulting from previous surgery, thereby leading to a drop in sensitivity to 50%. Even in a case previously reported by Kaori Seki et al. [29] wherein the patient had not undergone surgery, MIBI failed to detect a parathyroid tumor, which was thought to be related to the tumor’s small size [30, 31]. The blood flow and abnormal location of the parathyroid adenoma may affect the uptake and accumulation of radioactive substances [32]. These reasons may explain the negative finding of the cystic solid mass in the sheath of the left carotid artery in our patient on MIBI imaging. MIBI has also shown false-positive results [33–35] in other studies possibly due to radiopharmaceutical uptake of thymoma or lymphoma.

MIBI can also be positive for thyroid follicular lesions that may affect pHPT localization. According to reports, the mechanism of radiopharmaceutical uptake depends on the size of the tumor, the blood flow within the tumor, and the abundance of mitochondria in tumor cells [36, 37]. With the increase of blood flow and mitochondrial concentration, the degree of its absorption increases. Before this patient's first operation, right lower parathyroid mass uptake of MIBI was present, and the possibility of right lower parathyroid adenoma was considered. However, postoperative pathological report suggested a thyroid follicular adenoma. This may be related to the radiopharmaceutical uptake of this tumor cell in MIBI examination. As a result, failure of accurate lesion location in this patient suggested that in pHPT patients with possible thyroid follicular lesions, especially in those with suspected ectopic adenoma, the diagnosis and surgery should be cautious. US and MIBI may be not reliable imaging tools in this situation. Further precise lesion locating using other diagnostic methods is needed.

When US and MIBI are inconclusive, negative, or show a major ectopia in the mediastinum, CT or MRI is recommended, which is considered useful in these cases. However, previous reports have also showed that CT and MRI both had false-negative and false-positive results due to tumor’s size and localization (such as in the thymus) [38].

In sum, US and MIBI are the most common localization methods for pHPT. It can increase the diagnostic rate up to 90% when neck US is combined with MIBI in patients with non-operative pHPT. However, in cases with negative or discordant diagnosis by non-invasive methods or in some persistent or recurrent cases after surgery when the sensitivity of the above methods decreases, further examination needs to be considered.

### SVS—a sensitive and accurate method

Despite being an invasive technique, SVS has been considered a useful method [39]. SVS is a reliable method to quickly measure PTH by collecting blood samples through a venous catheter. Compared with the baseline, two fold increases in PTH is identified as a positive result [40]. The result depends on the PTH produced by the parathyroid gland but not its size. More specifically, as the sampling site approaches the lesion, the level of PTH increases. Although SVS is time-consuming, expensive, invasive, and carries the risk of infection [41], it has been shown to be more sensitive and accurate in localizing the lesion than MIBI, increasing the success rate of second surgery [28]. In recurrent or persistent hyperparathyroidism requiring second operation, SVS has been reported to have high sensitivity value, range from 75% to 94.7%, for the detection of the specific area of ectopic parathyroid tissue [42].

After SVS, the cystic solid mass in the sheath of the left carotid artery was highly suspected to be an ectopic parathyroid gland in our patient, which was negative by other non-invasive examination methods. The final pathological results revealed that it was an ectopic parathyroid adenoma after surgery. It is difficult to make a definitive diagnosis in some cases, and there may be false positives before surgery or insufficient exposure of the anterior cervical field during operation. These factors make it more necessary to perform an SVS examination before the operation. Intrathyroid parathyroid adenoma, a rather rare condition, is often difficult to be located non-invasively before surgery and is often missed as the parathyroid gland cannot be easily distinguished from thyroid nodules. Gofrit et al. [43] found that the sensitivity of US decreased from 87 to 64% due to the presence of thyroid nodules. If no parathyroid glands are found during surgery, a blinded lobectomy will be performed on the ipsilateral suspected lesion [44], which may cause more damage to patients. By providing the specific position of increased PTH secretion, SVS may help to reduce surgical time and complications. Therefore, SVS could be a useful preoperative localization method for this type of patient. However, in some specific situations that a pregnant woman with suspicion of pHPT, the situation can be even more complex and make the diagnosis even more difficult [45]. Due to the radiation's effects on the fetus, CT and MIBI should be avoided during pregnancy. For the same reason, SVS is not recommended for pregnant patients. Therefore, US remains the gold standard for diagnosis and location of pHPT in pregnant patients. Ectopic parathyroid adenoma and schwannoma.

The parathyroid glands are generally located near the thyroid gland, but there are exceptions; it is estimated that ectopic parathyroid glands may occur in 2% individuals [46]. The ectopic parathyroid which is not in the normal anatomical position can be found in intrathyroid, submandibular, mediastinal thymus, and carotid sheath according to published studies. In a meta-analysis including 7005 patients and 23,519 parathyroid glands, 15.9% parathyroid glands were in ectopic locations [47]. We reviewed and summarized a total of 41 cases of reoperation owing to ectopic parathyroid identified through SVS previously reported in several studies [3, 28, 39, 48–56] (Table 1).

**Table 1:** Summary of previously reported cases of reoperation caused by ectopic parathyroid identified through SVS

Authors	Year	Cases(n)	Venous localization	Pathology	Prognosis
**Onnicha **[3]	2020	1	in the left carotid sheath near the ipsilateral strap muscle	hyperplasias	cured
**Oliver **[48]	2012	2	in the thymus, in the carina, caudal of the aortic arch	adenoma (2/2)	cured (2/2)
**Janneke **[28]	2010	5	in the mediastinum (4/5), on the left side of the neck on the prevertebral fascia (1/5)	adenoma(2/5), hyperplasias (2/5), unknown (1/5)	cured (2/5), unknown (3/5)
**Neveen **[49]	2007	1	below the right lower pole of the thyroid gland	adenoma	cured
**M. Wiedmann **[50]	2007	1	in the submandibular area of the left jaw	adenoma	cured
**C M Ogilvie **[51]	2006	1	retro-oesophageal	carcinoma	cured
**E. ESTELLA **[39]	2003	6	posterior to apex of pleura and right recurrent laryngeal nerve at T1 level, in right superior thyroid, lying posteriorly over prevertebral fascia, in thymic parenchyma, junction right brachiocephalic vein and common carotid artery, abutting inferolateral and posterior aspect of left lower pole thyroid	adenoma (5/6), unknown (1/6)	hypercalcemia (1/6), permanent hypocalcemia (4/6), cured (1/6)
**Robert **[39]	2003	3	Right ectopic upper retroesophageal, Left lower carotid sheath, Right upper ectopic retroesophageal	adenoma (3/3)	cured (3/3)
**L. Morbois-Trabut **[52]	2002	2	in the mediastinum	adenoma(1/2), hyperplasias (1/2)	cured (2/2)
**Saky **[53]	2001	1	the right dome of the diaphragm	adenoma	cured
**Bengt **[54]	1994	16	in the mediastinum	adenoma(16/16)	cured (16/16)
**Akira **[55]	1991	1	in the upper mediastinum	adenoma	cured
**TADAICHI **[56]	1980	1	the right lateral wall of the esophagus	adenoma	cured

In our literature review, 60.98% (25/41) cases were in the mediastinum, 36.59% (15/41) were in the neck, and 2.44% (1/41) cases were in the right dome of the diaphragm. Of the 41 patients, only 39 showed postoperative pathology. Adenoma accounted for 87.18% (34/39), followed by 10.26% (4/39) hyperplasia and 2.56% (1/39) carcinoma. In all of these cases, the reasons for the failure of the previous operations were not described in detail or mentioned in the literature in 36 cases. The remaining five patients displayed negative US or MIBI results. In the population with persistent or recurrent pHPT, the possibility of ectopic parathyroid glands has been overlooked. Abnormally located glands often make it impossible for the surgeon to accurately locate the lesion, which accounts for 50% cases of unsuccessful parathyroid surgery.

Moreover, taking into account the characteristics of cervical schwannoma, parathyroid adenoma in the neck should be differentiated from cervical schwannoma. The common non-specific symptom of schwannoma, which may also cause hypercalcemia, is an isolated, slow-growing mass in the neck [57, 58]. Per Calcaterra et al.’s [59] study, more than one-third of schwannomas originate in the head and neck region. Hence, in consideration of the similar location and hypercalcemia, schwannoma needs to be differentiated from ectopic parathyroid adenoma.

Imaging methods such as US, MRI, and CT can be used for identification. However, the diagnosis of this disease, to a certain degree, is usually non-specific. In this case, the P-pHPT after surgery and the mass in the sheath of the left carotid artery which should be differentiated from schwannoma and ectopic parathyroid adenoma significantly increase the difficulty of diagnosis and reoperation. The successful diagnosis and resection of the lesion in this patient strongly indicated that in patients with P-pHPT and suspected ectopic parathyroid adenoma or schwannoma, SVS should be conducted for precise diagnosis and lesion location before planning the second operation.

## Conclusion

Although reoperation is difficult and complex, choosing the right examination can improve the success rate of parathyroid reoperation. We report a patient with P-pHPT in whom SVS accurately identified the ectopic parathyroid gland, while conventional non-invasive imaging studies failed to determine the exact location of the lesion. In pHPT patients accompanied with possible thyroid follicular lesions, lesion location according to noninvasive methods should be done cautiously before surgery. Patients with P-pHPT who require a reoperation, especially when the lesion needs differentiation from ectopic parathyroid adenoma and schwannoma, should undergo SVS as it can provide precise diagnosis and accurate positioning before the second operation.

## Data Availability

The data that support the findings of this study are available from the corresponding author upon reasonable request.

## Abbreviations

PTH: Parathyroid hormone level
US: Ultrasonography
CT: Computed tomography
MRI: Magnetic resonance imaging
MIBI: 99MTc-Sestamibi scintigraphy
SVS: Selective venous sampling
PHPT: Primary hyperparathyroidism
PET: Positron emission tomography
DXA: Dual X-ray absorptiometry
BMD: Bone mineral density
P-pHPT: Persistent primary hyperparathyroidism
